# Left ventricular mural thrombus and dual coronary embolization associated with hyperthyroid cardiomyopathy and atrial fibrillation: a case report

**DOI:** 10.1186/s12872-017-0565-7

**Published:** 2017-05-19

**Authors:** Guohui Liu, Ping Yang, Yuquan He

**Affiliations:** Department of Cardiology, China-Japan Union Hospital, Jilin University, Changchun, Jilin Province 130000 China

**Keywords:** Embolization, acute myocardial infarction, balloon angioplasty, atrial fibrillation

## Abstract

**Background:**

The majority of acute myocardial infarction (AMI) events are caused by thrombotic occlusion of the coronary artery, secondary to atherosclerotic plaque erosion or rupture. However, coronary embolism (CE), while rare, is being increasingly recognized as an important cause of AMI. We present the case of a patient with multi-site coronary artery embolization associated with hyperthyroid-related cardiomyopathy and atrial fibrillation.

**Case presentation:**

A 49-year-old female with a history of hyperthyroidism and atrial fibrillation (AF) was admitted to our hospital presenting with right upper limb pain and swelling. Initial transthoracic echocardiography demonstrated left ventricular apical mural thrombi and hyperthyroidism-induced cardiomyopathy. On the eighth day after admission, the patient developed sudden onset of severe chest pain and evidence of acute myocardial infarction (AMI). Emergency coronary angiography revealed multi-site coronary embolization of the left anterior descending artery and a large diagonal branch. Despite emergency thrombo-aspiration and balloon angioplasty, the patient went into ventricular fibrillation, from which she did not recover.

**Conclusion:**

Although rare, a fatal case of left ventricular thrombus and dual-vessel coronary embolism associated with hyperthyroid cardiomyopathy and atrial fibrillation is reported.

## Background

Coronary embolism (CE) is a rare cause of acute myocardial infarction (AMI). Multi-site CE involving two coronary arteries is even rarer. We present the fatal case of a patient who had dual coronary embolism with atrial fibrillation secondary to hyperthyroid cardiomyopathy.

## Case presentation 

**Fig. 1:**
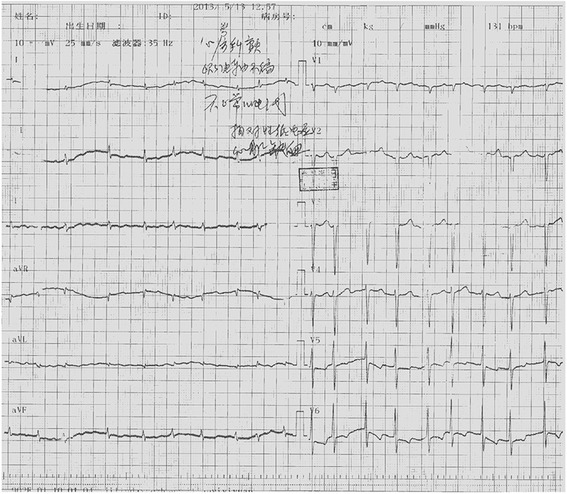
ECG shows AF and ST-segment depression and T-wave inversion on leads V5-V6

**Fig. 2:**
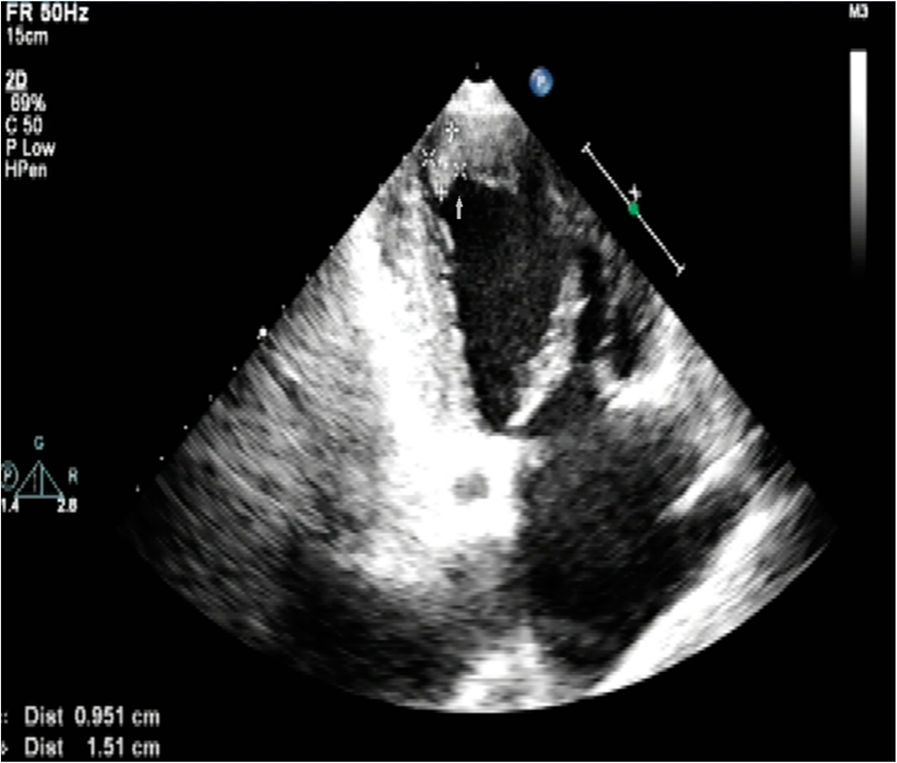
A thrombus of 15 × 9.5 mm is seen in the left ventricular apex

A 49-year-old Chinese female was admitted to the Department of Vascular Surgery on the 13th of May, 2013 presenting with right upper limb pain and swelling that had persisted for 10 days. Two years prior to admission, the patient was diagnosed with hyperthyroidism and persistent atrial fibrillation (AF). At admission, the patient had not been taking her anticoagulation or anti-platelet aggregation therapy for more than a year. Physical examination revealed a blood pressure of 98/63 mmHg and fast AF with ventricular beating rates in excess of 100 beats/min. Cardiac auscultation revealed variable intensity of first heart sound and a grade 2/6 tricuspid systolic murmur. Upon physical examination, the right upper limb was cold, pale and swollen, with positive tenderness and no palpable radial and brachial pulses. Electrocardiography (ECG) showed AF, ST-segment depression and T-wave inversion on leads V5-V6 (Fig. 1). Echocardiography revealed enlargement of right atrium (47 mm), left ventricular end-diastolic dilatation (60 mm), and diffuse left ventricular anterior wall hypokinesis with an ejection fraction (EF) of 28%. Significantly, a (mural or mobile) thrombus measuring 15 **×** 9.5 mm was present at the left ventricular apex. In addition, there was evidence of moderate mitral regurgitation, severe tricuspid regurgitation, and an elevated mean pulmonary arterial pressure (30 mmHg) (Fig. 2). Right upper limb vascular ultrasound showed thrombi in the right innominate vein, subclavian vein, internal jugular vein, axillary vein, brachial vein, and the proximal segment of right brachial artery. The chest radiograph showed an enlarged heart without pulmonary congestion.

Laboratory thyroid function test revealed thyroid-stimulating hormone level of 0.005 mIU/L, free T3 level of 9.71 pmol/L, and a free T4 level of 47.1 pmol/L, all of which are indicative of active hyperthyroidism. Liver and kidney function tests, routine blood coagulation assessment, and urine analysis were all normal.

The patient had a history of hyperthyroidism in the absence of other risk factors for heart failure, so hyperthyroid-related cardiomyopathy could be diagnosed. Meanwhile atrial fibrillation aggravated heart failure. Upon admission, the patient was treated with intravenous urokinase infusion (200,000 units daily) for the first 7 days of admission. The right upper limb pain and swelling gradually improved, and the radial and brachial pulses returned.

**Fig. 3:**
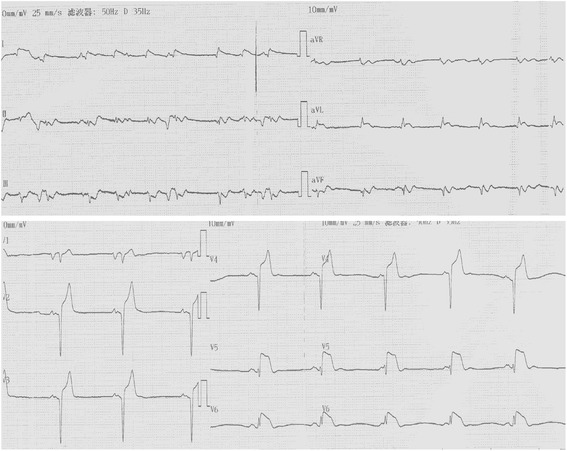
ECG shows sinus rhythms, convex-upward ST-segment elevation and T wave merge to form a large upright monophasic deflection on leads I, aVL, V1-V6

**Fig. 4:**
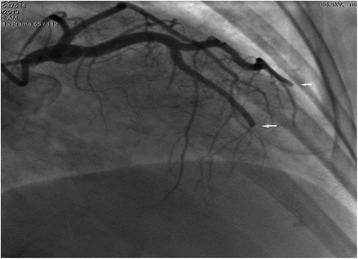
Coronary angiography shows occlusions in the distal ends of the LADCA and the first diagonal artery

**Fig. 5:**
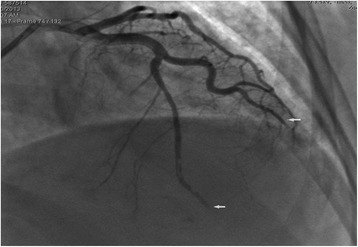
After PTCA, the thrombi moved to the distal ends of the LADCA and the first diagonal artery

**Fig. 6:**
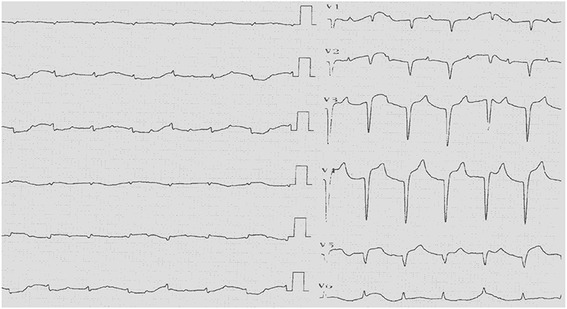
ST segments went downward on leads V1-V6 and QS waves appeared on leads V1-V5

However, on May 20, 2013, a week after admission, the patient complained of a sudden onset of severe central chest pain, which was not relieved by sublingual or intravenous nitroglycerine. The patient collapsed suddenly within 5 min of pain onset and ECG monitoring indicated ventricular fibrillation (VF) and convex-upward ST-segment elevation, and merging T wave in leads I, aVL, and V1-V6, consistent with anterior acute ST-elevation myocardial infarction (Fig. 3). Cardiopulmonary resuscitation (CPR) was immediately initiated and spontaneous circulation and respiration were restored. However, the patient remained unconscious and an emergency coronary angiography was performed. Left coronary angiography demonstrated abrupt ‘cut-off’ of the distal ends of both the left anterior descending (LAD) coronary artery and the first diagonal artery, consistent with embolization; the circumflex and right coronary arteries were normal (Fig. 4). Thrombus aspiration was performed using a 6-French Export Aspiration catheter (Medtronic; crossing profile, 0.068 in.), initially in the LAD coronary artery followed by the first diagonal artery. No thrombi or debris were aspirated. Therefore, balloon angioplasty was next performed using a 2.0 **×** 20 mm Ryujin balloon in the LAD coronary artery and the first diagonal artery occlusion sites. The balloon angioplasty caused the thrombi to migrate more distally in both the LAD and diagonal arterial branches (Fig. 5). The procedure was thus terminated and the patient was returned to the coronary care unit (CCU) with stable hemodynamics and persistent AF. Continuous intravenous infusion of tirofiban at 10 ml/h was initiated in the CCU. Subsequently, ECG showed resolution of ST segments elevation on leads V1-V6 (Fig. 6). Troponin I and creatine phosphokinase MB isoenzyme levels were 0.04 ng/ml (normal range: 0–0.04 ng/mL) and 95 U/L (normal range 0–16 U/L), respectively, 1 h after the onset of tirofiban treatment. Additional maintenance medical treatment regimen included dopamine and metaraminol administration. However, 10 h after attempted coronary recanalization therapy, the patient suffered a second VF cardiac arrest from which she failed to be resuscitated.

## Discussion and Conclusions

The majority of acute myocardial infarction (AMI) events are caused by thrombotic occlusion of coronary artery, secondary to atherosclerotic plaque erosion or rupture. However, coronary artery embolism (CE) is now recognized as an important non-atherosclerotic cause of AMI [1], with previous studies showing that 4 to 7% of AMI patients had non-atherosclerotic coronary arteries based on coronary angiography or autopsy findings [2, 3]. While rare, the prevalence of CE may currently be underestimated given the high risk of thromboembolic events in patients with prosthesis, rheumatic valvular diseases, infective endocarditis, cardiomyopathy, chronic AF, intra-cardiac shunts, tumors, and other hypercoagulable states [4, 5].

In the present report, we have highlighted a fatal case of a young female patient with hyperthyroidism-related cardiomyopathy and chronic AF who was non-compliant with her anti-coagulation therapy, leading to AMI due to multi-site coronary artery embolization. In addition to the coronary artery emboli, the patient had evidence of a right brachial arterial embolism. In reviewing the case, a mural thrombus discovered in the left ventricular apex was the likely source of the emboli.

In retrospect, detachment of thrombotic debris secondary to the urokinase thrombolytic therapy may have aggravated the multi-focal vascular embolization. In support of CE, coronary angiography showed no evidence of stenotic atherosclerotic plaque and the patient’s coronary arterial wall appeared smooth. The multi-site distal coronary occlusions seen during coronary angiography support coronary artery embolization as the likely cause for the patient’s sudden onset of AMI, precipitating the ventricular fibrillation that proved fatal. The incidence rate of thromboembolism caused by hyperthyroidism-related AF has been reported to vary from 8 to 40% [6, 7], with most AF-associated thrombi occurring in the left atrial appendage. In contrast, we observed thrombus formation in the left ventricular apex. This rare occurrence could be due to the patient being in a hypercoagulable state as evident by the concomitant thrombosis observed in her right upper limb veins. However, thrombotic risk factors, such as the presence of protein C or protein S, were not estimated in this case to confirm.

Although the patient was initially treated with warfarin anticoagulation therapy in accordance with the ACC/AHA guidelines that clearly recommend early anticoagulation therapy in such patients [8], she had unfortunately been non-compliant with her oral anticoagulation therapy for the past year. The decreased left ventricular EF, left ventricular anterior wall hypokinesis, and vortex formation following reduced apical blood flow could have predisposed this patient to left ventricular apical thrombosis.

As coronary artery embolism is relatively rare, consensus guidelines for its treatment are currently not available. Of the potential therapeutic approaches, triple anti-platelet therapy (aspirin, clopidogrel, IIb/IIIa receptor inhibitors) [9], intracoronary catheter aspiration [2], PTCA [10], stent implantation [11], and coronary arterial thrombectomy [12] have proven successful. Our case of CE-induced AMI was complicated by malignant arrhythmia and cardiac arrest at AMI onset, requiring immediate resuscitation and coronary reperfusion therapy by primary angioplasty. Furthermore, when right upper limb brachial embolism, venous thrombosis, and the presence of left ventricular thrombi was discovered upon admission, the decision was made to treat the patient with continuous urokinase infusion thrombolysis for a week and left ventricular thrombectomy was not performed. In retrospect, the latter procedure would have been beneficial. When the patient developed AMI diagnosed to be due to multi-site coronary artery embolization, manual catheter aspiration was attempted in both the LAD and the first diagonal coronary arteries. However, aspiration of the embolized thrombi was unsuccessful, presumably because the thrombi had fibrosed, making them larger than the lumen of the aspiration catheter and difficult to dissolve. Nevertheless, postoperative ECG revealed some resolution of the ST segment elevation. Since the thrombi that had migrated distally and the occluded coronary vessels were less than 2.25 mm in diameter, stent implantation was not performed. Lastly, plain balloon coronary angioplasty was unsuccessful in completely restoring coronary blood flow to a TIMI 3 grade. The patient eventually succumbed to sustained VF secondary to her AMI, despite active resuscitation and appropriate medical treatment. In retrospect, intra-aortic balloon pump could have been applied during the operation to enhance her coronary perfusion and, potentially, improve procedural success.

This case highlights the importance of anticoagulation therapy for thromboembolic prophylaxis in patients with hyperthyroidism-related cardiomyopathy and AF. Left ventricular thrombus formation and AMI caused by coronary artery embolism are rare, but could be fatal if not properly managed, as seen in this case. Treatment regimens should be based on the patient’s specific conditions and risk factors. Left ventricular thrombectomy could be considered when a large or mobile thrombus is evident. Coronary thrombo-aspiration, balloon dilation, stent implantation and coronary thrombectomy are therapeutic options when coronary artery embolization AMI is suspected. To treat distal emboli, there are three other methods that should be considered if aspiration fails: 1) Balloon dilation could make the thrombi deformable, which is beneficial to increase blood flow and cause the thrombi to migrate distally, potentially reducing myocardial infarct size; 2) 5 French catheter (5 in 6 catheter) may enable aspiration of larger thrombi than a typical aspiration catheter; and 3) Use of multiple guidewires winding to physically remove the thrombi may be another choice, though this method increases the risk of other side branch emboli. In conclusion, this case should remind us of the high-risk posed by the presence of cardiac thrombosis, with thrombi dislodgement potentially leading to coronary artery embolization and AMI. Early systemic anti-thrombosis therapy and patient compliance is necessary to mitigate both short- and long-term adverse outcomes, but more effective or novel approaches for CE treatment is warranted in the future.

## Abbreviations

AF: Atrial fibrillation
AMI: Acute myocardial infarction
CCU: Coronary care unit
CE: Coronary embolism
CPR: Cardiopulmonary resuscitation
ECG: Electrocardiogram
EF: Ejection fraction
LAD: Left anterior descending coronary artery
VF: Ventricular fibrillation
